# Supplementary material for: Modeling glioblastoma heterogeneity as a dynamic network of cell states
Source: Mol Syst Biol. 2021 Sep 16;17(9):e10105. doi: 10.15252/msb.202010105 (PMC8444284; doi:10.15252/msb.202010105)
Supplement: Supplementary file 6 — Source Data for Figure 5 [file MSB-17-e10105-s004.zip › Figure5A_sourcedata/GSEA_3017/nosigdb_stateD.GseaPreranked.1622795010397/RICHARDS_NOWAKOWSKI_SCIENCE_2017_IN_CTX_CGE1_UPREG.html]

Details for gene set RICHARDS\_NOWAKOWSKI\_SCIENCE\_2017\_IN\_CTX\_CGE1\_UPREG[GSEA]

|  || Dataset | state23017 |
| Phenotype | NoPhenotypeAvailable |
| Upregulated in class | na\_pos |
| GeneSet | RICHARDS\_NOWAKOWSKI\_SCIENCE\_2017\_IN\_CTX\_CGE1\_UPREG |
| Enrichment Score (ES) | 0.112351865 |
| Normalized Enrichment Score (NES) | 1.0580834 |
| Nominal p-value | 0.38 |
| FDR q-value | 0.5498231 |
| FWER p-Value | 0.966 |
Table: GSEA Results Summary

  

Fig 1: Enrichment plot: RICHARDS\_NOWAKOWSKI\_SCIENCE\_2017\_IN\_CTX\_CGE1\_UPREG      
 Profile of the Running ES Score & Positions of GeneSet Members on the Rank Ordered List

  

| PROBE | GENE SYMBOL | GENE\_TITLE | RANK IN GENE LIST | RANK METRIC SCORE | RUNNING ES | CORE ENRICHMENT || 1 | SOX11 |  |  | 6 | 0.578 | 0.0288 | Yes |
| 2 | BTG1 |  |  | 50 | 0.350 | 0.0153 | Yes |
| 3 | CALM3 |  |  | 82 | 0.308 | 0.0088 | Yes |
| 4 | SOX4 |  |  | 91 | 0.298 | 0.0198 | Yes |
| 5 | CHD7 |  |  | 116 | 0.278 | 0.0170 | Yes |
| 6 | ZNF22 |  |  | 119 | 0.276 | 0.0315 | Yes |
| 7 | NSA2 |  |  | 143 | 0.261 | 0.0285 | Yes |
| 8 | LEPROTL1 |  |  | 145 | 0.260 | 0.0429 | Yes |
| 9 | MEF2A |  |  | 163 | -0.251 | 0.0440 | Yes |
| 10 | PHF3 |  |  | 169 | -0.251 | 0.0546 | Yes |
| 11 | PPP1R12B |  |  | 174 | -0.252 | 0.0661 | Yes |
| 12 | NNT |  |  | 188 | -0.253 | 0.0705 | Yes |
| 13 | POU2F1 |  |  | 194 | -0.253 | 0.0813 | Yes |
| 14 | GNAQ |  |  | 201 | -0.253 | 0.0913 | Yes |
| 15 | EIF4G3 |  |  | 205 | -0.254 | 0.1036 | Yes |
| 16 | NAP1L4 |  |  | 215 | -0.255 | 0.1114 | Yes |
| 17 | FAM172A |  |  | 250 | -0.260 | 0.0996 | Yes |
| 18 | NF1 |  |  | 254 | -0.260 | 0.1124 | Yes |
| 19 | EEA1 |  |  | 291 | -0.263 | 0.0993 | No |
| 20 | ZEB2 |  |  | 317 | -0.265 | 0.0949 | No |
| 21 | SUB1 |  |  | 371 | -0.270 | 0.0689 | No |
| 22 | USP33 |  |  | 375 | -0.271 | 0.0823 | No |
| 23 | RIF1 |  |  | 403 | -0.274 | 0.0769 | No |
| 24 | PNRC2 |  |  | 406 | -0.274 | 0.0912 | No |
| 25 | RBM5 |  |  | 423 | -0.276 | 0.0946 | No |
| 26 | ATM |  |  | 460 | -0.280 | 0.0826 | No |
| 27 | CASD1 |  |  | 463 | -0.281 | 0.0973 | No |
| 28 | DLX1 |  |  | 490 | -0.284 | 0.0933 | No |
| 29 | RUFY3 |  |  | 539 | -0.290 | 0.0723 | No |
| 30 | ARFGAP3 |  |  | 545 | -0.290 | 0.0852 | No |
| 31 | CCDC93 |  |  | 610 | -0.303 | 0.0524 | No |
| 32 | DCAF7 |  |  | 649 | -0.310 | 0.0405 | No |
| 33 | PPP1R9A |  |  | 675 | -0.315 | 0.0391 | No |
| 34 | CEP350 |  |  | 710 | -0.321 | 0.0309 | No |
| 35 | DPYSL3 |  |  | 722 | -0.323 | 0.0410 | No |
| 36 | ST8SIA5 |  |  | 737 | -0.327 | 0.0490 | No |
| 37 | JAKMIP2 |  |  | 837 | -0.354 | -0.0084 | No |
| 38 | RND3 |  |  | 853 | -0.358 | 0.0005 | No |
| 39 | GAS2L3 |  |  | 856 | -0.359 | 0.0198 | No |
| 40 | PDE4DIP |  |  | 866 | -0.362 | 0.0337 | No |
| 41 | RTN1 |  |  | 906 | -0.376 | 0.0248 | No |
| 42 | XPO1 |  |  | 967 | -0.400 | 0.0008 | No |
| 43 | MEG3 |  |  | 975 | -0.404 | 0.0188 | No |
| 44 | DCLK2 |  |  | 986 | -0.408 | 0.0346 | No |
| 45 | CTNND2 |  |  | 992 | -0.411 | 0.0545 | No |
| 46 | CDH2 |  |  | 1007 | -0.418 | 0.0677 | No |
| 47 | PCDH9 |  |  | 1019 | -0.427 | 0.0838 | No |
| 48 | SRGAP2C |  |  | 1029 | -0.432 | 0.1018 | No |
| 49 | MPC1 |  |  | 1086 | -0.462 | 0.0846 | No |
| 50 | APLP1 |  |  | 1249 | -0.700 | -0.0024 | No |
| 51 | CHL1 |  |  | 1300 | -0.991 | 0.0157 | No |
Table: GSEA details [plain text format]

  

Fig 2: RICHARDS\_NOWAKOWSKI\_SCIENCE\_2017\_IN\_CTX\_CGE1\_UPREG: Random ES distribution      
 Gene set null distribution of ES for **RICHARDS\_NOWAKOWSKI\_SCIENCE\_2017\_IN\_CTX\_CGE1\_UPREG**
